# Early Onset of Wilson's Disease and Possible Role of Disease-Modifying Genes: A Case Report and Literature Review

**DOI:** 10.1155/crhe/3815089

**Published:** 2024-11-25

**Authors:** Alessandro La Rosa, Angela Elvira Covone, Domenico Coviello, Serena Arrigo, Jacopo Ferro, Paolo Gandullia, Annalisa Madeo

**Affiliations:** ^1^Paediatric Gastroenterology and Digestive Endoscopy Unit, IRCCS Istituto Giannina Gaslini, Genoa, Italy; ^2^Laboratory of Human Genetics, IRCCS Istituto Giannina Gaslini, Genoa, Italy; ^3^Department of Laboratory Medicine, Division of Anatomic Pathology, IRCCS Istituto Giannina Gaslini, Genoa, Italy

**Keywords:** ATP7B, disease-modifying genes, early-onset, HFE, Wilson's disease, zinc acetate

## Abstract

Wilson's disease (WD) is a rare autosomal recessive disorder caused by mutations in the ATP7B gene, resulting in copper accumulation. Symptoms rarely appear before the age of 5, almost never before 3. The phenotypic variability of WD suggests the presence of modifying factors, making early diagnosis challenging. We present a case of symptomatic WD in a toddler, emphasizing the importance of considering WD in differential diagnoses and exploring genetic modifiers influencing disease onset. Clinical and laboratory assessments, including liver biopsy, were performed on a 4.2-year-old boy presenting with hypertransaminasemia and mild hepatomegaly. Histological evaluation revealed chronic hepatitis with fibrosis and severe steatosis, indicating long-standing active disease. Genetic analysis identified a missense variant and a 15-nucleotide deletion in the 5′ UTR promoter region of the ATP7B gene, confirming the WD diagnosis. Additionally, homozygosity for the HFE H63D variant was detected, with transferrin saturations at the upper limit of normal. The patient's clinical management included a trial of D-penicillamine, discontinued due to side effects, followed by successful zinc acetate therapy. This case underscores the consideration of WD in the differential diagnosis of toddlers. The Ferenci-Leipzig score remains a valid diagnostic tool for WD even in the presence of a single ATP7B variant, although extended genetic analysis should still be considered. Normal ceruloplasmin levels do not rule out WD. Environmental, epigenetic, and genetic factors appear to influence the WD phenotype; HFE variants may act as modifiers given the link between iron and copper homeostasis, possibly explaining the early symptomatic onset in our patient.

**Summary**

  Take-Home Message: Consider WD in toddlers with consistent symptoms after excluding other causes, exploring modifying factors that deepen the disease phenotype; the Ferenci-Leipzig score remains reliable even with only one ATP7B variant, suggesting the need for extended genetic analysis. Treatment with first-line zinc acetate seems to reverse clinical manifestations.

## 1. Background and Aims

Wilson's disease (WD) is a rare pathology whose clinical and laboratory manifestations are a direct consequence of copper overload, initially in the liver and subsequently in other tissues such as the brain, eye, and kidney. WD is an autosomal recessive hereditary disorder due to mutations of the ATP7B gene, which encodes for a hepatocytic transporter that allows copper incorporation into ceruloplasmin and the efflux of copper in excess through the bile flow [1].

The phenotypic expression of the disease is highly variable and probably influenced by other factors acting as clinical and biochemical modifiers; this concept is increasingly gaining ground in the scientific community in the era of omics sciences [2].

Indeed, WD clinical manifestations can first appear between 5 and 35 years, rarely under 5 and almost never before 3 years [3], and it is evident that no genetic variant acts alone.

Herein, we present the case of a patient with early symptomatic onset of WD, with the aim of underlining the importance of taking into account this disease even in toddlers if clinical suspicion is high and/or once other more frequent causes have been excluded. Furthermore, we would like to speculate on the possible action of modifying genes that could impact in the disease onset, thereby suggesting their inclusion in the diagnostic and genetic analysis processes.

## 2. Materials and Methods

No ethical approval was required. Written consent for blood sample collection, experimentation, and publication was obtained from the parents of the patient. The patient was evaluated almost monthly until diagnosis and the start of treatment, then after 1 month, and subsequently every 6 months. The patient is still on a semiannual follow-up.

### 2.1. Histological Evaluation

Liver biopsy was obtained by the Menghini technique under ultrasound guidance. For histological examination, paraffin-embedded 4 *μ*m sections were stained with haematoxylin and eosin, Pas for glycogen, Pas-diastasis, trichromic for fibrosis assessment, Gomori for reticular fibers, and Perls Prussian Blue for iron deposits. Moreover, immunohistochemical reactions for CD3, CD20, CD68 (PGM1), cytokeratins 7 and 19, for inflammatory infiltrates characterization and ductular reaction evaluation, and collagen IV were performed.

After a description of the histological lesions observed, METAVIR staging system to assess parenchymal fibrosis and Ishak grading system to assess necroinflammatory activity were performed.

### 2.2. Genetic Analysis

Genomic DNA was extracted from peripheral blood using Symphony automatic extractor (Qiagen).

*Whole-Exome-Sequencing* (WES) was performed on genomic coding regions and exon-intron junctions (5 nucleotides) using the kit for Clinical Exome CES_v2: 4490 genes (SOPHiA Genetics) on Miseq platform (Illumina). For the purpose of our analysis, patient data underwent a comprehensive analysis of 140 genes associated with hereditary liver disease and WD as suggested by the Human Phenotype Ontology browser (HPO):

ABCB11, ABCB4, ABCC2, ABCD3, ABCG8, ACADM, ACADS, ACADVL, AGL, AKR1D1, ALDOB, ALG1, ALG3, ALG6, ALG8, ALG9, ALMS1, AMACR, AP1S1, ARG1, ARHGAP31, ASL, ASS1, ATP7B, ATP8B1, BAAT, BCS1L, CC2D2A, CFTR, CLDN1, COG1, COG2, COG3, COG4, COG5, COG6, COG7, COG8, CPT1A, CPT2, CYP27A1, CYP7A1, CYP7B1, DGUOK, DHCR7, DKC1, DLD, DOCK6, EIF2AK3, ENG, EPHX1, ETFA, ETFB, ETFDH, FAH, FBP1, G6PC, GALE, GALK1, GALT, GBA, GBE1, HADH, HADHA, HADHB, HAMP, HFE, HNF1B, HSD3B7, JAG1, LIPA, LRP5, MKS1, MPI, MPV17, NBAS, NEK8, NOTCH1, NOTCH2, NPC1, NPC2, NPHP1, NPHP3, NPHP4, NR1H4, OFD1, OTC∗, PEX1, PEX10, PEX11B, PEX12, PEX13, PEX14, PEX16, PEX19, PEX2, PEX26, PEX3, PEX5, PEX6, PEX7, PGM1, PHKA2, PHKB, PHKG2, PKD1, PKD2, PKHD1, PMM2, POLG, PRKCSH, PYGL, RBPJ, RPGRIP1L, SEC63, SERPINA1, SLC22A5, SLC25A13, SLC25A20, SLC27A5, SLC2A2, SLC30A10, SLC37A4, SLC40A1, SLC7A7, SLCO1B1, SLCO1B3, SMPD1, SP110, TALDO1, TFR2, TJP2, TMEM216, TMEM67, TRMU, UGT1A1, UROS, UTP4, VIPAS39, VPS33B.

The minimum target read depth was 20X with optimal coverage ≥ 98%, only one gene (∗) had a lower coverage equal to 90% while, our gene ATP7B had maximum coverage (100%). Data filtration and interpretation were carried out using SOPHiA DDM: v5.9.1.1. Reference databases used were the Human Reference Genome hg19 and the Human Gene Mutation Database dbSNP15. Furthermore, we used the Integrative Genomic Viewer (IGV) software for visualization of sequence data and variant calls.

Pathogenicity, population frequency and residue conservation of putative germline variants were evaluated according to approved guidelines by the American College of Medical Genetics (ACMG), ClinVar [4, 5] and bioinformatic tools from public databases (Polyphen2, SIFT, Mutation Taster, GnomAD, GERP, and others) supported by SOPHiA DDM and Varsome. The results of this analysis showed a missense variant in the gene ATP7B (OMIM 606882) defined as follows: chr13: 52532674-C-T; ATP7B (NM_000053.4): c.2128G > A; p.(Gly710Ser); rs137853285, in exon 8. This variant, in the heterozygous state in the proband, is considered pathogenic by the literature with a frequency of 0.0016% in the global population and the gene ATP7B is considered strongly associated to WD (# 277900) with autosomal recessive (AR) inheritance model. This variant was validated by Sanger sequencing in the proband and his parents with the purpose of analyzing the segregation within the family and resulted inherited from the mother.

*Whole-Genome-Sequencing* (WGS) was performed on genomic DNA of the proband and his parents using Novaseq 6000 platform (Illumina), Human Reference Genome hg38 and uniform 30X coverage referred to the proband. Trio analysis and interpretation were carried out using Geneyx software [6].

### 2.3. Validation and Segregation Analysis by Sanger Sequencing

**Figure 1:**
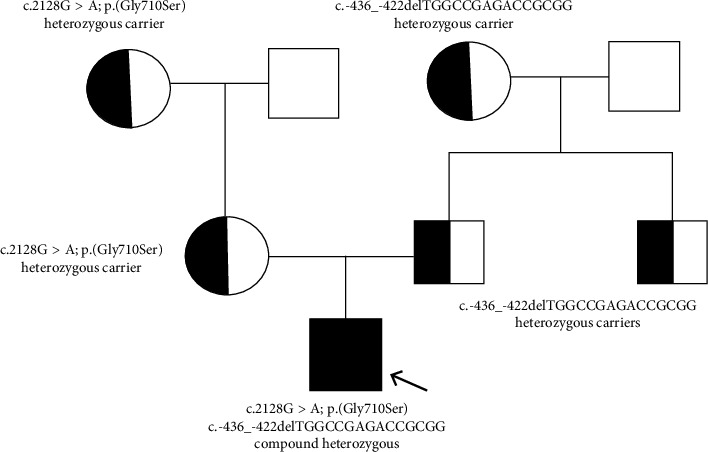
Pedigree: half‐filled diagrams are carriers; filled‐in diagram is carrier of both variants in a compound heterozygosity form.

Following familial request, we extended the study of both ATP7B variants to other family members for a better segregation analysis of the mutations (Figure 1).

Sanger sequencing was performed for validation and segregation analysis of both the missense and the 15-nucleotide deletion variant using BigDye Cycle Sequencing Kit (Applied Biosystems). The following primers are showed in the 5′ to 3′ direction:

ATP7B-exon 8-F: GAGATTTGTTTACTGAAGGAG; ATP7B-exon 8-R:CCTGTGGACAGTAGTCCTC; ATP7B-5′UTR-F: CAGCGCAGAGCGGACCCGA; ATP7B-5′UTR-R: GGAGTGCCCACCCTGGAAC.

**Figure 2:**
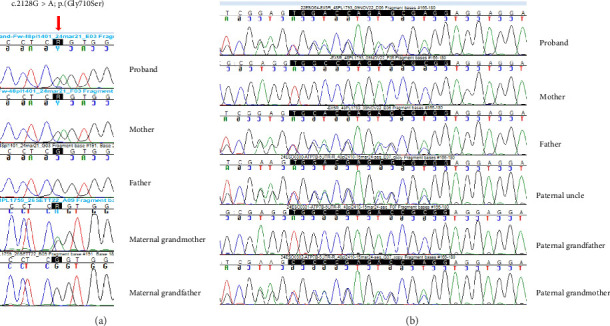
(a) Validation by Sanger sequencing of the missense variant c.2128G > A shows the variant allele (arrow) in heterozygous state in the proband, his mother and his maternal grandmother while, the father and the maternal grandfather show homozygosity for the reference allele. (b) Validation by Sanger sequencing of the deletion c.-436_-422delTGGCCGAGACCGCGG (highlighted by the black bar): the mother and the paternal grandfather show homozygosity for the reference allele and the correct reading frame; the proband, his father, the paternal uncle and paternal grandmother show heterozygosity for this variant that causes a reading frameshift (the sequence is shown in direction right to left).

The results of Sanger sequencing are described in Figures 2(a) and 2(b).

## 3. Case Report

A 4.2-years-old male child underwent CT-scan and brain MRI evaluation for the study of a head soft swelling discovered at the age of 2. Images demonstrated the presence of area of craniolacunia of the left fronto-parietal passage resulting in a slight outward prominence and enlarged periencephalic subarachnoid spaces of uncertain clinical significance, in absence of other lesions. At that time, blood tests were performed and an unexpected elevation of aspartate- and alanine-aminotransferase was found (AST 104 and ALT 203 U/L respectively - n.v. < 40 U/L). An autoimmune hepatitis was excluded since autoantibodies ANA, ASMA, AMA-M2, M2-3E, Sp100, PML, gp210, LKM-1, LC-1, ALS/LP, Ro-52 tested negative.

At the age of 4.6, the patient was admitted to the local hospital for an episode of macrohematuria. Lab tests showed AST 116 U/L, ALT 207 U/L, gamma-glutamyl-transpeptidase (GGT) 112 U/L, total bilirubin 0.52 mg/dL, ceruloplasmin 21.9 mg/dL (n.v. 20–60 mg/dL) and total serum copper at the lower limits (71 *μ*g/dL - n.v. 80–155 *μ*g/dL). An abdominal ultrasound bared a mild hepatomegaly with hyperechoic parenchyma, in absence of focal lesions and normal biliary tract anatomy, and a small calculus (4 mm) in a calyx of the left kidney, indicated as possible cause of urothelial bleeding.

At 4.7-years-old, the patient was hospitalized for the first time in Our Department for further evaluation about the moderate persisting hypertransaminasemia, mild hepatic steatosis and recent macrohematuria.

Blood tests confirmed an increase in the hepatic cytolysis indices, ceruloplasmin at the lower limits (19 mg/dL), hemoglobin 12.7 g/dL, ferritin 122 ng/mL (n.v. 20–200 *μ*g/dL), serum iron 158 *μ*g/dL (n.v. 70–140 *μ*g/dL), transferrin 336 mg/dL (n.v. 203–360 mg/dL), transferrin saturation 33% (n.v. 15%–45%), antibodies for celiac disease and fecal calprotectin were negative, lysosomal acid lipase activity was within the normal range.

**Figure 3:**
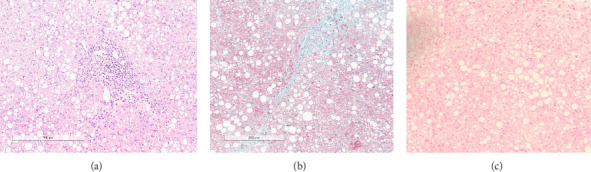
(a) Liver biopsy showed a pattern of diffuse macrovesicular steatosis and expansion of portal spaces with mild mixed inflammatory infiltrates; (b) histochemical investigation by Masson's Trichrome showed the presence of fibrosis even with porto-portal septa formation; (c) pearls Prussian blue histochemical staining showed no iron deposits, both in the hepatocytes and in the Kupfer cells.

An echo-guided liver biopsy was planned, and histological evaluation pointed out a picture of chronic hepatitis with portal, interface and lobular inflammation, portal-periportal fibrosis (Ishak score 3, METAVIR F2) and severe panlobular micro- and macrovesicular steatosis, no iron deposition was found (Figure 3); intrahepatic copper was 637 microg/g (n.v. 0–200) hence WD was suspected. A 24 h urinary collection demonstrated an elevated calcium output of 5.2 mg/kg/day equal to 153 mg/L (n.v. ≤ 100 mg/L) and high urinary copper excretion, 206 *μ*g/24 h (n.v. < 40 *μ*g/24 h), strengthening the diagnostic suspicion of WD.

Thereafter, at the age of 5, the patient was evaluated by ophthalmologist without finding any eye abnormalities and a genetic analysis employing a next-generation-sequence (NGS) liver panel was performed.

The analysis of the ATP7B gene highlighted in the proband a missense variant c.2128G > A (p.Gly710Ser) in heterozygosity, inherited from the mother. An Array-CGH 180k Agilent array (Human Genome CGH Microarray, Agilent Technologies, Santa Clara, USA) was then performed resulting negative for microdeletion in the 13q14.3 region (ATP7B gene). In addition, a missense mutation in the HFE gene c.187C > G; p.(His63Asp) in homozygosity was pointed out by the NGS panel.

Since the NGS panel failed to identify ATP7B biallelic mutations, WGS was performed.

According to the modified Ferenci-Leipzig score [7], a clinical diagnosis of WD was stated, and at the age of 5.8, the patient started D-penicillamine therapy with a dose-escalation program to reach 20 mg/kg/day and limit side effects.

The patient was admitted to the emergency room complaining fever, abdominal pain and an itchy erythematous rash, 1-week after the beginning of the treatment. Blood exams were consistent with an acute cholestatic hepatitis (AST 773 U/L, ALT 1178 U/L, GGT 380 U/L, LDH 656 mg/dL, total bilirubin 2.15 mg/dL, direct bilirubin 1.86 mg/dL). Assuming a possible adverse event due to D-penicillamine, the treatment was discontinued. Meanwhile, viral serologies were performed and then resulted positive for adenovirus, suggesting the possibility that symptoms could be due to the infection.

Hence, once recovered, treatment with D-penicillamine was resumed but, the patient experienced onset of fever, nausea, and malar rash again. Symptoms and temporal link were suggestive of drug hypersensitivity. So, treatment was stopped and zinc acetate 25 mg twice a day was started with good tolerance.

One week after the beginning of the new therapy, a clear improvement in blood tests was demonstrated (Table 1). Elastography showed a picture of mild/absent fibrosis (F0-F1, stiffness 6.5 kPa, IQR/med 16.2%).

WGS highlighted the presence of a 15-nucleotide deletion variant in the 5′ untranslated (UTR) promoter region of the ATP7B gene (c.-436_-422del) in the proband, inherited from the father, defined as follows: chr13:52,011,759:c.-436_-422delTGGCCGAGACCGCGG; rs1484840087, considered pathogenic by the literature [8]. This result, together with the previous known missense mutation inherited from the mother, allowed the molecular confirmation of WD in the proband due to a compound heterozygosity of two pathogenic variants in the ATP7B gene with AR inheritance model.

Currently, the patient is 7 years and has been on zinc acetate therapy for 1 year with good compliance and optimal disease response, presenting at the last blood test AST 43 U/L, ALT 55 U/L, and GGT 21 U/L. Despite the treatment, the kidney stone increased in size up to 8 mm, therefore patient underwent retrograde lithotripsy with benefit and no post-operative complications.

At the time this report is being redacted, the patient developed no neurological symptoms.

## 4. Discussion

We present a case of early onset symptomatic WD. It is known that the damage caused by copper intoxication is additional and linked to the progressive overload of this metal in the liver, bloodstream, eyes, and central nervous system. Thus, symptoms rarely appear before the age of five, with an average age at onset of 13.2 years, according to literature data [9].

The patient came to our attention 6 months after the first symptoms appeared, and the diagnosis of WD was made approximately 11 months later. This delay includes the time taken for copper evaluation on histology, which was performed in an external laboratory, as well as the technical time required to obtain the genetic test report. However, this timeline aligns with the literature, which indicates that the average delay between the onset of symptoms and the diagnosis of WD is about 1.8 to 2.7 years. This delay is particularly concerning because the symptoms of WD are often nonspecific and can mimic other disorders [10, 11].

At the age of 4, patient's liver histological exam revealed a moderately active chronic liver disease (Ishak score 3, METAVIR F2), the patient also presented nephrocalcinosis with macrohematuria and increased periencephalic CSF spaces, all described as clinical manifestations of WD in childhood [12–14].

There is no univocal genotype-phenotype correlation in WD; indeed clinical manifestations are variable even within the same family. This seems to be partially explained by the role of environmental, epigenetic, and genetic factors [15].

Hereditary hemochromatosis (HH) is a disease characterized by iron accumulation due to hepcidin deficiency [16]. Age at onset and HH phenotype are partially related to the genetic mutation and the hepcidin residual activity, requiring usually further concomitant factors to make iron overload clinically significant [17]. Moreover, gender has a pathogenetic role and males are at higher risk [18]. Four types of HH have been described of which type 1, due to HFE gene mutations, is the most frequent. HFE encodes for a membrane protein coupled to transferrin receptor 1 (TfR1) whose activity is to regulate the hepatocytes expression of hepcidin based upon the serum transferrin concentration. The most common pathogenic HFE variant is the C282Y resulting in significant iron overload [19]. Second in order of frequency is the H63D variant: patients homozygous or heterozygous for H63D are not at risk of HH but may have increased serum iron and transferrin levels [19, 20].

Accordingly, our patient is homozygous for the H63D mutation and showed transferrin in the upper quartiles of normal range and serum iron concentration slightly above the upper limit for the age (Table 1).

Homeostasis of iron and copper in mammals are linked [21]; ceruloplasmin uses its ferroxidase activity to convert iron from the ferrous (Fe(II)) to the ferric (Fe(III)) form, necessary step to bind and transport iron by transferrin. As a result, in hereditary aceruloplasminemia patients have low levels of plasma iron and of transferrin saturation with systemic iron excess [22]. Likewise, copper deficiency is the cause of iron-resistant anemia in adulthood [22]. Hence, concomitant HFE mutations in subjects with WD may contribute to modify the phenotypic expression [15], resulting in compound copper and iron overload [23]. By the way, in literature there are some case series describing HFE gene mutations effect on WD clinical manifestation, suggesting that the HFE variants may contribute to the iron overload, already present in WD therefore, acting as negative modifiers, but none describe the histological picture [24–28]. In individuals with the H63D mutation, iron can accumulate in the liver but typically does not reach clinically significant levels or cause notable fibrosis or cirrhosis unless combined with other conditions [29].

Here, we present a case of WD rather than early HH. Therefore, the absence of iron overload on Pearls Prussian Blue histochemical staining does not exclude a possible role of the HFE variant in disrupting copper metabolism, which may explain the presence of moderate fibrosis on histology and consistently high transferrin saturation levels for age [29]. Since our patient has both HFE and ATP7B mutations, we assume that his early symptomatic disease onset and the moderate histological liver damage could be due to the negative modifying action of the HFE gene on WD phenotype.

Even if patient's urinary zinc levels, under zinc acetate therapy at 25 mg twice a day, were below the recommended range, target levels of serum zinc and 24 h urinary copper excretion were achieved (Table 1) [9]. Therefore, at 7 years old, it was decided not to modify the zinc acetate posology to avoid potential over-treatment leading to further iron overload due to copper deficiency [30, 31] in a subject genetically predisposed to iron accumulation. Furthermore, even if first-line zinc monotherapy is not unequivocally indicated in symptomatic patients [9], data from our patient after 1 year of treatment demonstrate optimal tolerability and good efficacy, consistent with other case reports and series available in the literature [32, 33].

Interestingly, even though patient disease activity was already moderate at the onset, the ceruloplasmin levels were always around the lower limit (20 mg/dL). Hypoceruloplasminemia, particularly if < 14 mg/dL, is certainly an element of strong suspicion for WD with an estimated average sensitivity of 95% if it is dosed in subject older than 3 years. In other words, 5% of people with WD could result false negatives (FN) if only serum ceruloplasmin dosage as screening test is used; 5% of FN is an acceptable value for a screening method, but not negligible [9]. Therefore, ESPGHAN position paper recommends to dose both serum ceruloplasmin and urine copper excretion on 24-h collection as first tiers exams in case of suspected WD, in order to increase diagnostic sensitivity and specificity. Indeed, in our case, albeit ceruloplasmin values were borderline, a frankly pathological cupruria was found (>100 *μ*g/24 h). The borderline ceruloplasmin levels documented in our patient may be influenced by the ATP7B genotype. Specifically, the G710S missense mutation identified in this case has been classified as a “transport competent/trafficking defective” class of WD mutation [34]. This classification indicates that the mutation impairs the biliary pole trafficking of the ATPase while allowing some residual channel function, which could potentially explain the borderline ceruloplasmin levels observed.

The modified Ferenci-Leipzig score allows to diagnose WD using clinical and laboratory items, even if only one ATP7B gene pathogenic variant has been detected. This is important since it permits to start the de-coppering treatment earlier, therefore reducing the incipient damage due to copper progressive overload. Nowadays, more than 500 pathogenic mutations of ATP7B have been described and exome analysis is able to identify both mutated alleles only in 95% of affected subjects. In the remaining cases, small deletions/duplications, copy number variants (CNVs) in UTR and other noncoding regions may be undetected with this technique [9]. Hence, in case of strong clinical-laboratory suspicion it is advisable to extend the genetic analysis employing Array-CGH and/or WGS to identify pathogenic variants in the ATP7B gene, thus providing an adequate genetic counseling and screening of relatives.

In our case, this extended molecular analysis identified a deletion of 15 nucleotides in the 5′ UTR promoter region of the ATP7B gene, allowing us to confirm the patient's diagnosis from a molecular point of view. Interestingly, the 15 bp deletion within the ATP7B promoter region is the most common molecular defect in WD patients of Sardinian descent and the fact that in our family the paternal grandmother is of Sardinian descent once again indicates that this mutation most likely originated from a common ancestor and therefore resulted from a founder effect [7]. This case report has several limitations, beginning with the nature of the article itself, which describes a single patient. However, we believe that the history of our patient should suggest some reflections: first, the importance of taking into account WD in differential diagnosis also in toddlers, since this disease could be already symptomatic during the first years of life and it benefits from an adequate early treatment [35].

Furthermore, we confirm in a real-world experience the reliability of Ferenci-Leipzig score for diagnosis of WD even in the presence of a single ATP7B variant, recommending in any case to extend the genetic analysis in order to identify rare mutations or describe new ones, particularly in the noncoding regions of the gene [36]. Ceruloplasmin is undoubtedly a useful disease biomarker for screening, but it does not have a 100% sensitivity. Therefore, normal ceruloplasmin levels do not exclude at all WD especially in cases of strong clinical suspicion in which we remind you to extend the laboratory tests according to ESPGHAN Position Paper recommendations [9].

Finally, it is known that WD has a protean clinical expression, and environmental, epigenetic, and genetic factors seem to affect it. HFE could play a role as WD phenotype modifier for the intrinsic correlation of iron and copper homeostasis, probably justifying the early WD symptomatic onset in our patient [15, 24, 25, 37].

## Figures and Tables

**Table 1:** Patient hematological findings at baseline (4 y 3 mo), during the diagnostic work-up, and after therapy administration.

	4 y 3 mo	4 y 8 mo	4 y 9 mo	4 y 10 mo	5 y 0 mo	5 y 6 mo	5 y 8 mo	5 y 11 mo	6 y 0 mo	6 y 1 mo	6 y 7 mo	7 y 1 mo
Hb (12.5–14.8 g/dL)			12.7	13.0	12.7	13.5	12.8	11.9	10.3	13.2		12.6
Coombs test (Dir/Indir)				Neg/Neg								
AST (0–40 U/L)	104	116	157	212	163	114	172	150	99	58	47	43
ALT (0–40 U/L)	203		266	367	290	249	341	562	161	80	87	55
Gamma-GT (11–50 U/L)		122	117	148	113	122	133	319	85	56	40	21
Total bilirubin (0–1 mg/dL)		0.5	0.5	0.4	0.8			0.8	0.2	0.5	0.3	0.4
Ceruloplasmin (20–60 mg/dL)		21.9	19.0	21.0	17.0		21.0					
Serum total copper (80–155 *μ*g/dL)		71				78					82	53
Ferritin (20–200 ng/mL)			122	134							104	56
Iron (70–140 *μ*g/dL)			158	168			119			180	84	148
Transferrin (203–360 mg/dL)			336	319			336			344	311	307
Sat (transferrin) (15%–45%)			33%	37%			24%			36%	19%	33%
Urinary copper (15–70 *μ*g/24 h)						206	114.2			37.7	59.4	44.4
Serum zinc (68–107 *μ*g/dL)										183	186	167
Urinary zinc (0.15–1.2 mg/24 h)										0.5	1.2	1.0
		Episode of macrohematuria			ATP7B analysis found 1 missense mutation + HFE H63D/H63D variant	Ferenci-Leipzig score >4: WD clinical diagnosis		Start of D-penicillamine therapy and 1st AE	2nd D-penicillamine AE: stop and switch to Zinc acetate	One week after Zinc acetate begins		One year on Zinc acetate therapy

Abbreviations: Dir/Indir, direct and indirect coombs test; Hb, hemoglobin; mo, months; Sat, saturation; y, years.

## Data Availability

The data that support the findings of this study are available on request from the corresponding author. The data are not publicly available due to privacy or ethical restrictions.
